# The Effects of Breviscapine Injection on Hypertension in Hypertension-Induced Renal Damage Patients: A Systematic Review and a Meta-Analysis

**DOI:** 10.3389/fphar.2019.00118

**Published:** 2019-02-21

**Authors:** Lihua Wu, Yanhua Gao, Shufei Zhang, Zhuyuan Fang

**Affiliations:** ^1^Affiliated Hospital of Nanjing University of Chinese Medicine, Nanjing, China; ^2^Department of Pediatrics, Pizhou City Hospital of Traditional Chinese Medicine, Pi Zhou, China; ^3^Institute of Hypertension, Affiliated Hospital of Nanjing University of Chinese Medicine, Nanjing, China

**Keywords:** breviscapine injection, hypertension, systematic review, meta-analysis, effects

## Abstract

**Background:** Breviscapine (Dengzhanhua) injection has been wildly used in clinical treatment for cerebral infarction, cardiovascular disease, diabetic nephropathy, renal impairment of essential hypertension and stroke in China. Breviscapine injection and antihypertensive drugs combination therapy is supposed to be beneficial for hypertension-induced renal damage patients.

**Objectives:** To evaluate the beneficial and adverse effects of breviscapine injection on hypertension in hypertension-induced renal damage patients, an extensive meta-analysis was performed.

**Methods:** We searched PubMed, the Cochrane Library, Embase, CNKI, Sino Med, VIP, and Wanfang Data for relevant literature. The timeframe of retrieval was set from the founding date of each database to September 28, 2018.

**Results:** Fourteen papers were included in this study. The quality of all the studies included was determined to be low. All studies were conducted with Chinese populations. Meta-analysis showed that, compared with single-use antihypertensive drugs, using breviscapine injection in combination with antihypertensive drugs to treat hypertension in hypertension-induced renal damage patients can reduce 24-h urinary total protein (24 h UTP) [WMD = −0.04, 95% CI (−0.05, −0.02), *P* ≤ 0.001], but does not lower systolic blood pressure (SBP) [WMD = −1.02, 95% CI (−2.88, 0.84), *P* = 0.281] or diastolic blood pressure (DBP) [WMD = −0.21, 95% CI (−1.71, 1.29), *P* = 0.786] more effectively. There was also no statistically significant difference in adverse events between experimental groups and control groups.

**Conclusion:** Breviscapine injection, in combination with antihypertensive drugs, appears to be more effective in improving the 24 h UTP, but overall have no effect on improving the blood pressure in hypertension-induced renal damage patients. Moderate dose of breviscapine injection (10 ml) may have effects on reducing blood pressure in hypertension-induced renal damage patients but high doses of breviscapine injection (20 ml) may increase blood pressure by subgroup analysis. However, the evidence of methodological quality and sample sizes is weak, and thus, further standardized research is required.

## Introduction

Hypertension, defined as systolic blood pressure (SBP) ≥140 mm Hg or diastolic blood pressure (DBP) ≥90 mm Hg, is a risk factor for stroke, coronary artery disease, heart failure, peripheral vascular disease, and chronic kidney disease (CKD) (Lewington et al., 2002; Forouzanfar et al., 2017). Elevated blood pressure (BP) was the leading global contributor to premature death in 2015, accounting for almost 10 million deaths and over 200 million disability-adjusted life years (Forouzanfar et al., 2017). The main factors leading to the development of hypertension-induced renal damage include: (1) inappropriately elevated sympathetic nervous activity (SNA) (Joles and Koomans, 2004); (2) activation of the renin-angiotensin-aldosterone system (RAAS) (Grisk and Rettig, 2004); (3) increased arterial stiffness (Safar et al., 2001); (4) genetic susceptibility (Freedman et al., 2009); (5) impaired salt and water excretion by the kidney (ALLHAT Officers Coordinators for the ALLHAT Collaborative Research Group, 2002). Hypertensive kidney disease is the second leading cause of end-stage renal disease (ESRD) after diabetes mellitus (USRDS, 2018; Williams et al., 2018). In Europe, according to the European Dialysis and Transplant Association registry, hypertension-induced renal damage is accounted for 12% of new patients starting renal replacement therapy. However, the reported incidence varies among different countries, with France, Italy and United Kingdom, reporting in 25, 17, and 6.1%, with both Japanese and Chinese reporting in 6 and 7%, respectively (Fervenza et al., 2017). The diagnosis of hypertension-induced renal damage is based on the finding of reduced renal function and/or the detection of albuminuria (≥300 mg/d, or ≥300 mg/g albuminuria to-creatinine ratio in the first morning void). CKD is classified according to estimated glomerular filtration rate (eGFR), calculated by the 2009 CKD-Epidemiology Collaboration formula (Levey et al., 2009). Current evidence suggests that in patients with CKD, BP should be lowered to <140/90 mmHg and toward 130/80 mmHg. The combination of renin-angiotensin system blockers with calcium channel blockers (CCB) or diuretics should be used to achieve recommended blood pressure targets in CKD (Whelton et al., 2018; Williams et al., 2018). A recent meta-analysis have shown that BP lowering significantly reduced ESRD in patients with CKD, but only in patients with albuminuria, and had no beneficial effect on cardiovascular events (Lv et al., 2013). In a large retrospective cohort containing 398419 treated hypertensive patients, the nadir SBP and DBP for the lowest risk of ESRD and mortality were 137 and 71 mmHg, respectively, with a clear increase in mortality risk at SBP <120 mmHg (Sim et al., 2014). The evidence with respect to BP targets in patients with CKD is complex.

Breviscapine (Dengzhanhua) injection is extracted from *Erigeron breviscapus (Vant.), Erigeron breviscapus* also known as herba erigerontis or lamp chrysanthemum, is a traditional Chinese herb that has been in use for more than 600 years, found in Yunnan, Sichuan, Guizhou, and other southwest provinces of China. Breviscapine, as a purified flavonoid extract from this species, was first isolated by Zhang et al. (1988). Breviscapine mainly contains scutellarin (4′,5,6,7-tetrahydroxyflavone-7-O-glucuronide) and apigenin-7-O-glucuronide (Gao et al., 2017). Studies have shown that breviscapine has significant effects on vasodilation; inhibition platelet aggregation, scavenging free radicals, also has a protective effects on myocardial and endothelial structures because of its anti-inflammatory effects, and improve microcirculation; protection against ischemia/reperfusion (I/R); anticoagulation and antithrombosis; reduction of smooth muscle cell migration and proliferation; anticardiac remodeling;antiarrhythmia, and reduction of blood lipids (Jia et al., 2008; Wang et al., 2008, 2010, 2015). Breviscapine has been demonstrated to possess a number of pharmacological functions in addition to its hemodynamic effects; it has been concluded that breviscapine can relax norepinephrine-induced vasoconstriction in a concentration-dependent manner (Zheng et al., 1998); it has been linked to the scavenging of oxygen free radicals, decreasing the expressions ofintercellular adhesion molecule-1 protein in the myocardium and increasing the activities of Na(+)-K(+)-ATPase, Mg(2+)-ATPase, Ca(2+)-ATPase in the myocardial mitochondria (Jia et al., 2008); it has been reported that breviscapine could prevent thrombosis and platelet aggregation and improve the characteristics of haemorheology by restricting the ADP-induced platelet aggregation rate (Song et al., 2011); it could obviously inhibit the proliferation of vascular smooth muscle cell (VSMC) and may prevent atherosclerosis, and the mechanism may be realized partly by regulating NF-κB activity of VSMC (Pang et al., 2004); it has been reported to serve as an anti-oxidative stress agent and a protein kinase C (PKC) inhibitor, can inhibits the glycogen synthase kinase 3β (GSK3β) signaling pathway to promote neurobehavioral function following neurotrauma, and can improve renal function and reduce urinary micro-albuminuria (He et al., 2012; Liu et al., 2016; Jiang et al., 2017; Wang et al., 2018). In the light of these pharmacological activities, an injection preparation of breviscapine (a traditional Chinese patent medicine) has been wildly used in clinical treatment for cerebral infarction, cardiovascular disease, diabetic nephropathy, renal impairment of essential hypertension and stroke in China (Yang and Li, 2007; Liu et al., 2016; Gao et al., 2017; Wang et al., 2018).

However, in the past decades, although numerous clinical trials have been published analyzing the beneficial effects of breviscapine injection as an adjunctive therapy for hypertension-induced renal damage. However, there is no critical appraisal of the evidence on whether breviscapine injection as a complementary therapy could decrease BP for hypertension-induced renal damage patients. Therefore, we did a systematic review and meta-analysis to provide more reliable evidence on the effect of breviscapine injection on BP and other key outcomes.

## Materials and Methods

### Database and Search Strategies

We designed our systematic review and meta-analysis in accordance with the guidelines of the 2009 Preferred Reporting Items for Systematic Reviews and Meta-analysis (PRISMA) statement. Foreign databases searched included PubMed, Embase, and the Cochrane Library. Chinese databases included the China National Knowledge Infrastructure (CNKI), China Biology Medicine Disc (Sino Med), the VIP information resource integration service platform (VIP), and the Wanfang Data knowledge service platform (Wanfang Data). The retrieval scheme was mainly based on a combination of subject words and free words. The searched words were: “Dengzhanhua,” “Dengzhanhua preparations,” “Dengzhanhua Zhusheye,” “Zhusheyong Dengzhanhua,” “Gaoxueya Shenbing,” “Gaoxueya Shenyan” and “Gaoxueya Shenshunhai,” while the searched English words were: “Breviscapine,” “Breviscapine Injection,” “BVP,” “Hypertension-induced renal damage,” “Hypertensive Nephropathy,” “Hypertension, Renal,” “Hypertensive Kidney Lesion,” and “Hypertensive Renal Damage” (see Supplementary Tables 1, 2 for search strategy). The retrieval language was not limited, and the timeframe of the retrieval was from the founding date of each database to September 28, 2018. There was language limitation. Manual searches of relevant literature supplemented the electronic search.

### Inclusion Criteria

Randomized controlled trials (RCTs) that use breviscapine injection in combination with antihypertensive drugs to treat hypertension-induced renal damage, regardless of blinding, were included in this study. Language was also not restricted as to minimize publication bias. There were no serious organic diseases or complications in the selected cases. The diagnosis of hypertension-induced renal damage is based on the finding of reduced renal function and/or the detection of albuminuria. CKD is classified according to eGFR, calculated by the 2009 CKD–Epidemiologyn Collaboration formula (Levey et al., 2009). Hypertension was defined as SBP ≥ 140 mmHg or DBP ≥ 90 mmHg were based on the Chinese Guidelines for the Prevention and Treatment of Hypertension (2010), and the Eight Joint National Commission (JNC8). We did not limit inclusion based on age, sex, case source, disease course, hypertensive classification, or antihypertensives. Experimental group: Breviscapine Injection combined with antihypertensive drugs[captopril, amlodipine, lisinopril, benazapril, losartan potassium, felodipine, nifedipine, etc. (medication dose, medication time and frequency, and treatment course)]. Control group: Antihypertensive drugs included captopril, amlodipine, lisinopril, benazapril, losartan potassium, felodipine, nifedipine, etc. (medication dose, medication time and frequency, and treatment course). The age, sex, and other baseline conditions of the research objects were well-matched.

### Exclusion Criteria

Studies were excluded if they were: (1) clinical trials from which no relevant data could be extracted; (2) studies that were published repeatedly; (3) populated with inconsistent baseline information (age, sex, case source, disease course, hypertensive classification, or antihypertensive drugs); (4) systematic review, important data report, and case reports; no reply from corresponding authors such that further data could not be obtained; and (5) therapeutic measures failing to meet the predetermined inclusion criteria.

### Data Extraction

Primary outcomes were SBP and DBP. Secondary outcomes included 24-h urinary total protein (24 h UTP) and adverse effects. Two evaluators independently performed a search according to the search strategy, and preliminary screening was based on independent topics and abstracts of the search results, excluding obviously unqualified documents. A full-text methodology screening was conducted on the literature that might meet the inclusion criteria, and corresponding authors were contacted when there was incomplete information. Then, the studies were cross-checked by two evaluators. Any disagreement on the conclusion of two evaluators was resolved by discussion. If such disagreement could not be resolved through discussion, final judgment and arbitration was made by a third party. Extracted contents included authors' names, year of publication, number of samples, intervention, course of treatment, and observed indicators.

### Quality Evaluation

The investigators simultaneously evaluated the bias risk of the included studies based on the “risk of bias” evaluation tool in the Cochrane Handbook for Systematic Reviews (Review Manager, 2014) of interventions and relevant assessment guideline regulations. This risk evaluation tool contains seven items: (1) random sequence generation; (2) allocation concealment; (3) blinding of the participants and personnel; (4) blinding of the outcome data; (5) incomplete outcome data; (6) selective reporting; and (7) other bias., and were evaluated as having a “high risk of bias,” “low risk of bias,” or “unclear risk of bias” according to assessment criteria.

### Data Analysis

(1) Stata 14.0 software was used to perform the statistical analysis for the meta-analysis (Higgins and Thompson, 2002). (2) Select effect size: if an index of the included documents is a binary variable, the curative effect analysis statistics can be represented by relative risk (RR) and expressed by its confidence interval (CI); mean difference (MD) and 95% CI were used to represent continuous changes. (3) Homogeneity test: test the variation degree of original research results and clearly include the degree of homogeneity of the experiment. (4) Meta-analysis: according to the result of the heterogeneity test, *P* ≥ 0.05 and *I*^2^ < 50 indicate that the results have good agreement and that the fixed effect model may be used. *P* < 0.05 and *I*^2^ ≥ 50 suggest that the heterogeneity of the results cannot be ignored. If the included studies still have clinical significance, the random effects model may be used. (5) Sensitivity analysis: in those meta-analysis of the comprehensive factors combined with multiple outcomes, possible anomalous studies were ruled out before reevaluation. The results were compared with those of meta-analysis before the exclusion to determine how the excluded studies would influence the combined effect size and the stability of meta-analysis. If there is little difference between the two results, then the sensitivity of the results is relatively low, and the results are stable, indicating high credibility. (6) Subgroup analysis: subgroup analysis was conducted on some indexes with high heterogeneity. For events in which quantitative synthesis was impossible and events with very low incidence, qualitative evaluation may be based on the description. In this study, Stata 14.0 software was used to conduct a sensitivity analysis and subgroup analysis and to create a sensitivity analysis chart. Publication bias occurs when positive data in similar research papers with statistical significance are more likely to be published in journals. This situation is hard to control. The funnel plot method is often used to detect publication bias. Egger's test was performed to detect publication bias in the outcome measures, and a funnel plot was drawn. If a large publication bias was found in a particular research index, the exact reason should be identified.

## Results

### Search Results

**Figure 1:**
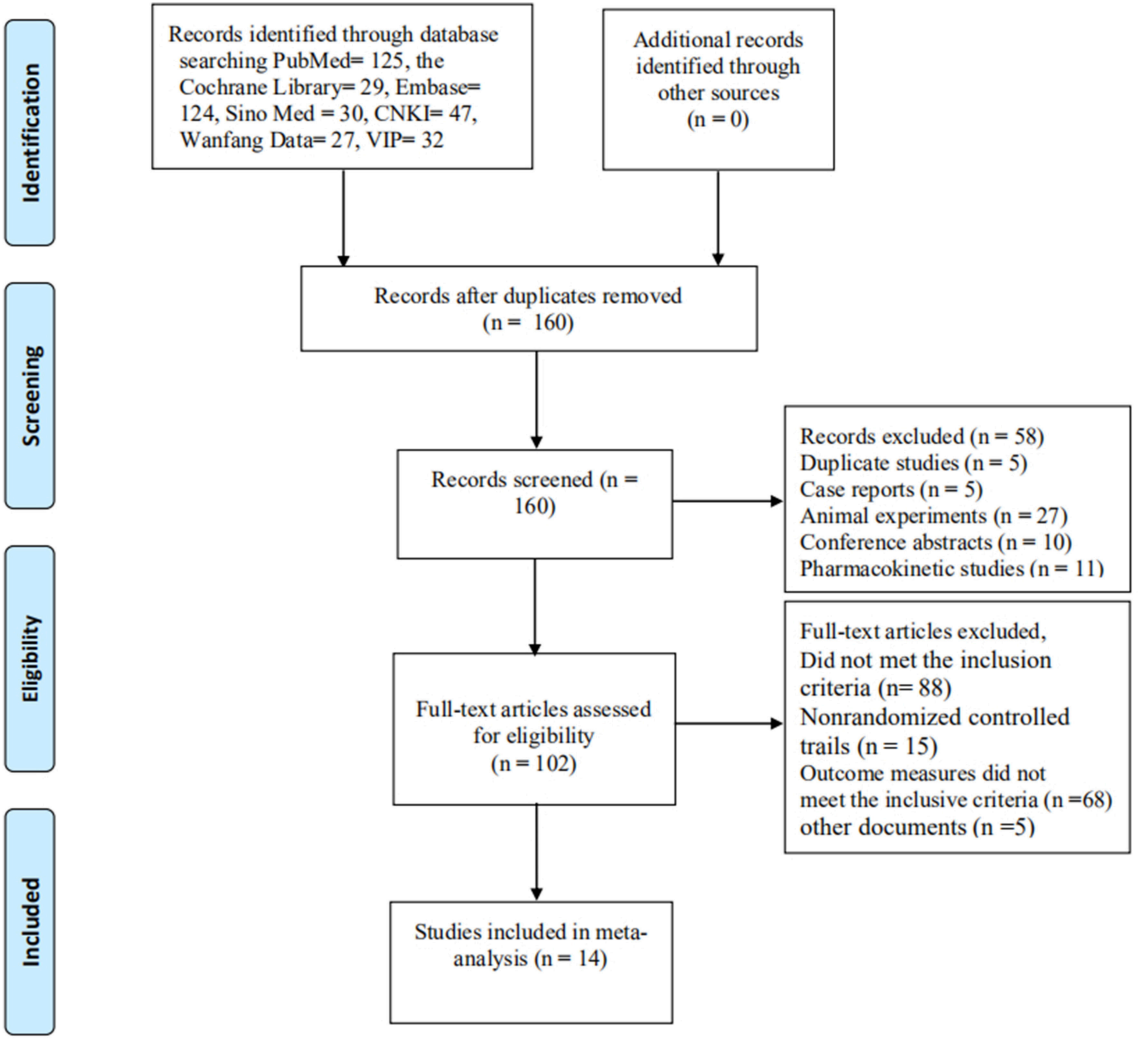
Flowchart of the process for literature retrieval.

A total of 414 documents [the Cochrane Library (*n* = 29), PubMed (*n* = 125), Embase (*n* = 124), Sino Med (*n* = 30), CNKI (*n* = 47), Wanfang Data (*n* = 27), and VIP (*n* = 32)] met the data collection and search strategy conditions. NoteExpress, a professional document management software, was employed to check for duplication of the 414 obtained articles that met the relevance requirement. The majority of these trials were excluded because some papers were found in more than one database and some included irrelevant titles and abstracts. Only 160 studies were retrieved. Following a review of the titles and abstracts, several studies were excluded, and only 102 studies remained. Five trials were excluded because of duplicated publications. Twenty-seven trials were excluded for being animal studies, and twenty-six trials were excluded for being non-clinical trials, including case reports, pharmacokinetic studies, and conference abstracts. Eighty-eight out of the remaining 102 articles were excluded based on the inclusion criteria, leaving 14 RCTs to be reviewed in Figure 1.

### Study Characteristics

There were 14 randomized controlled trials (Zhang et al., 2004; Wei and Tan, 2005; Ren and Wu, 2006; Zheng, 2006; Chen et al., 2008; Ye, 2009, 2013; He and Gen, 2010; Liu, 2012; Wang and Lan, 2012; Huang et al., 2013; Qiao, 2015; Zhao and Dong, 2016; Ma, 2018) that were included in the present research involving 1,170 patients (593 in the research group and 577 in the control group). These 14 RCTs are summarized in Tables 1, 2.

**Table 1:** Characteristics of the included studies.

**Study**	**Sample size (T/C)**	**Sex M/F**	**Age (years) range, mean**	**Country**	**Diagnosis standards**	**Intervention**	**Control**	**Course of treatment**	**Outcomes**	**Adverse reactions**
Zhang et al., 2004	94 (47/47)	T: 18/16, C: 17/13	T: 60 ± 8, C: 60 ± 8	China	GMY (1999WHO–ISH)	Breviscapine injection (20 ml, ivgtt, qd) + control	Lisinopril (5–10 mg, po, tid), 25mg, tid	2 weeks	24 h UTP, SBP, DBP, B2M, TC, TG, LDL, LDH –C, Hct, PAGT, Fib	T: 2 cases of dry cough, C: 3 cases of dry cough
Wei and Tan, 2005	76 (40/36)	T: 30/10, C: 28/8	T: 63.5, C: 64.3	China	GMY (1999WHO–ISH)	Breviscapine injection (20 mL, ivgtt, qd) + control	Amlodipine (5 mg, po, qd)+ Captopril (25 mg, po, tid)	4 weeks	24 h UTP, BUN, Scr, blood B2M, Urine B2M	T: 1 showed mild facial flushing during infusion, C: None
Ren and Wu, 2006	60 (30/30)	T: 21/9, C: 22/8	T: 44 −75, C: 43–74	China	CGMY (2004)	Breviscapine injection (30 mL, ivgtt, qd) + control	Captopril (25–75 mg, po, qd) or Nifedipine (20–60 mg, po, qd)	4 weeks	Clinical efficacy, 24 h UTP, BUN, Scr,	T: 2 cases of dry cough, C: 1 cases of mild head inflation
Zheng, 2006	72 (37/35)	T: 22/13, C: 23/14	T: 41–73, C: 42–71	China	GMY (1999WHO–ISH)	Breviscapine injection (30 mL, ivgtt, qd) + control	Captopril (25–75 mg, po, bid or tid) or Nifedipine (10–60 mg, po, bid or tid)	4 weeks	24 h UTP, BUN,Scr,	T: 2 cases of cough, C: 1 cases of mild head inflation
Chen et al., 2008	57 (28/29)	T: 19/9, C: 20/9	T:45.6 ± 20.3, C: 46.4 ± 21.1	China	CGMY (2004)	Breviscapine injection (20 mL, ivgtt, qd) + control	Benazapril (no details)	4 weeks	Clinical efficacy, 24 h UTP, BUN, Scr„ Ccr	T: 2 cases of head swelling, dizziness during infusion, C: 1 case of cough
Ye, 2009	75 (40/35)	T: 24/16, C: 20/15	T: 47.3 ± 18.2, C: 46.9 ± 16.1	China	GMY (1999WHO–ISH) and CGMY (2005)	Breviscapine injection (5 mL, ivgtt, qd) + control	Losartan Potassium (100 mg, po, qd)	4 weeks	24 h UTP, Scr„ Ccr, Urine B2M, SBP, DBP	T: 1 case of dizziness, C: None
He and Gen, 2010	168 (84/84)	T: 49/35, C: 48/36	T: 69 ± 11, C: 68 ± 11	China	CGMY (2005)	Breviscapine injection (20 mL, ivgtt, qd) + control	Captopril (12.5–50 mg, po, tid)	30 days	Clinical efficacy, SBP, DBP, 24 h UTP, Scr, BUN	T: 5 cases of dry cough, C: 3 cases of dry cough
Wang and Lan, 2012	103 (52/51)	Unclear	T: 62.4 ± 4.8, C: 62.4 ± 4.8	China	GMY (1999WHO–ISH) and Nephrology (Haiyan Wang)	Breviscapine injection (10 mL, ivgtt, qd) + control	Lisinopril (20 mg, po, qd)	30 days	Clinical efficacy, SBP, DBP, 24 h UTP, Scr, BUN	T: 1 cases of dry cough, C: 2 cases of dry cough
Liu, 2012	50 (26/24)	T: 12/14, C: 10/14	T: 55 ± 4, C: 53 ± 5	China	GMY (1999WHO–ISH) and CGMY (2005)	Breviscapine injection (10 mL, ivgtt, qd) + control	Benazapril (10 mg, po, bid)	2 weeks	SBP, DBP, Urinary microalbumin	Not reported
Huang et al., 2013	63 (33/30)	T: 24/9, C: 19/11	Unclear	China	CGMY (2004)	Breviscapine injection (20 mL, ivgtt, qd) + control	Felodipine (5 mg, po, qd)+ Aspirin (100 mg, po, qd)	4 weeks	Clinical efficacy, SBP, DBP, 24 h UTP, Scr, BUN, TC, TG, blood glucose	T: 2 cases of limb skin redness, C:None.
Ye, 2013	48 (24/24)	T: 16/8, C: 14/10	T: 53.0 ± 9.3, C: 49.0 ± 11.6	China	CGMY (2010)	Breviscapine injection (12 mL, ivgtt, qd) + control	Antihypertensive Drugs (no details) + Prostaglandin E injection (2 ml, ivgtt, qd)	30 days	Clinical efficacy, 24 h UTP, TC, TG, Scr, BUN,	Not reported
Qiao, 2015	158 (79/79)	Unclear	T: 48.01 ± 3.15, C: 48.01 ± 3.15	China	GMY (1999WHO–ISH) and CGMY (2005)	Breviscapine injection (5 mL, ivgtt, qd) + control	Captopril (25–75 mg, po, tid) or Nifedipine (10–60 mg, po, tid)	4 weeks	Clinical efficacy, SBP, DBP, 224 h UTP, Scr, BUN	Not reported
Zhao and Dong, 2016	80 40/40)	Unclear	T: 52.5 ± 7.1, C: 52.5 ± 7.1	China	CGMY (2010)	Breviscapine injection (5 mL, ivgtt, qd) + control	Benazapril (5 mg, po, qd)	4 weeks	Col–IV, LN, P III P, ET−1, MMP−9, NO, EILA, EISA, VOI, SBP, DBP	None
Ma, 2018	66 (33/33)	T: 19/14, C: 20/13	T: 48.00 ± 3.14, C: 48.99 ± 2.98	China	GMY (1999WHO–ISH) and CGMY (2005)	Breviscapine injection (5 mL, ivgtt, qd) + control	Losartan Potassium (100 mg, po, qd)	4 weeks	Urine B2M, SBP, DBP, 24 h UTP, Scr, Ccr	Not reported

*T, treatment group; C, control group; GMY (1999 WHO-ISH), The 1999 WHO-ISH Guidelines for the Management of Hypertension; CGMY (2004), 2004 Chinese Guidelines for the Management of Hypertension; CGMY (2010), 2010 Chinese Guidelines for the Management of Hypertension; CGMY (2014), 2014 Chinese Guidelines for the Management of Hypertension; Guiding Principles (2002), Guiding Principles of Clinical Research on New Drugs of Chinese Medicines (2002); Scr, serum creatinine; BUN, blood urea nitrogen; Ccr, creatinine clearance rate; 24 h UTP, 24-h urinary total protein; B2M, beta-2-microglobulin; SBP, systolic blood pressure; DBP, diastolic blood pressure; TC, total cholesterol; TG, total triglycerides. PAGT, platelet aggregation rate; Fib, fibrinogen;Hct, hematocrit; LDL, low-density lipoprotein; LDH-C, lactate dehydrogenase C; Col-IV, collagen IV, LN, laminin; P III P, procollagen-III-peptide; ET-1, endothelin 1; MMP-9, matrix metalloproteinase-9; EILA, elasticity index of large arter; EISA, elasticity index of small artery; VOI, vascular overload index*.

**Table 2:** Study characteristics: Effect of breviscapine injection on blood pressure and 24–hour urinary total protein in patients with hypertension-induced renal damage.

**Study**	**Systolic blood pressure (mmHg)**		**Diastolic blood pressure (mmHg)**		**Study**	**24–h urinary total protein (g/24 h)**	
	**Baseline**	**After intervention**	**Baseline**	**After intervention**		**Baseline**	**After intervention**
Zhang et al., 2004	T: 160.6 ± 9.70	T: 132.8 ± 7.8	T: 97.6 ± 6.4	T: 83.1 ± 4.2	Zhang et al., 2004	T: 14.39 ± 1.67	T: 4.77 ± 1.03
	C: 159.1 ± 8.9	C: 131.9 ± 7.6	C: 96.6 ± 6.1	C: 82.2 ± 4.6		C: 14.27 ± 1.55	C: 9.5 ± 1.14
Ye, 2009	T: 151.6 ± 10.4	T: 123.3 ± 7.3	T: 95.3 ± 7.6	T: 82.1 ± 6.5	Wei and Tan, 2005	T: 0.351 ± 0.022	T: 0.232 ± 0.019
	C: 152.7 ± 8.8	C: 124.2 ± 6.1	C: 94.6 ± 6.1	C: 80.9 ± 7.2		C: 0.364 ± 0.023	C: 0.280 ± 0.037
He and Gen, 2010	T: 160 ± 8.4	T: 135.4 ± 7.6	T: 99.5 ± 8.1	T: 84.5 ± 4.2	Ren and Wu, 2006	T: 1.54 ± 0.82	T: 1.30 ± 0.86
	C: 162 ± 9.1	C: 132.2 ± 5.3	C: 98.3 ± 9.2	C: 82.1 ±4.6		C: 1.55 ± 0.78	C: 1.54 ± 0.89
Wang and Lan, 2012	T: 161.5 ± 8.8	T: 130.6 ± 7.5	T: 98.8 ± 7.2	T: 82.1 ± 4.4	Zheng, 2006	T: 1.46 ± 1.22	T: 1.31 ± 0.90
	C: 160.6 ± 9.4	C: 131.8 ± 8.4	C: 98.9 ± 7.3	C: 83.2 ± 4.5		C: 1.45 ± 1.22	C: 1.44 ± 1.20
Liu, 2012	T: 159 ± 9	T: 132 ± 4	T: 93 ± 6	T: 82 ± 5	Chen et al., 2008	T: 6.8 ± 3.5	T: 2.1 ± 1.1
	C: 16 2± 8	C: 136 ± 5	C:92 ± 6	C:84 ± 3		C: 6.2 ± 2.2	C:3.0 ± 1.3
Huang et al., 2013	T: 148.5 ± 11.2	T: 132.2 ± 11.1	T: 89.5 ± 8.2	T: 82.4 ± 5.2	Ye, 2009	T: 0.102 ± 0.027	T: 0.031 ± 0.024
	C: 146.6 ± 12.3	C:130.4 ± 9.9	C:87.5 ± 7.8	C:81.3 ± 6.2		C: 0.098 ± 0.026	C: 0.043 ± 0.02
Qiao, 2015	T: 168.12 ± 9.36	T:126.32 ± 2.36	T: 96.87 ± 5.46	T: 76.98 ± 4.99	He and Gen, 2010	T: 0.153 ± 0.051	T: 0.095 ± 0.031
	C: 167.73 ± 9.32	C:127.30 ± 2.31	C:97.86 ± 5.33	C:77.18 ± 5.19		C: 0.154 ± 0.054	C: 0.123 ± 0.043
Zhao and Dong, 2016	T: 160.45 ± 19.18	T: 128.24 ± 15.64	T: 105.49 ± 12.18	T: 83.72 ± 9.46	Wang and Lan, 2012	T: 1.52 ± 0.80	T: 1.11 ± 0.87
	C: 160.41 ± 19.24	C: 142.53 ± 16.80	C: 102.52 ± 12.34	C: 92.24 ± 10.05		C: 1.54 ± 0.79	C: 1.26 ± 0.90
Ma, 2018	T: 151.58 ± 10.36	T: 123.21 ± 7.26	T: 95.26 ± 7.62	T: 82.12 ± 6.54	Huang et al., 2013	T: 0.101 ± 0.027	T: 0.030 ± 0.023
	C: 152.65 ± 8.84	C: 124.15 ± 6.11	C: 94.62 ± 6.11	C: 80.91 ± 7.24		C: 0.098 ± 0.026	C:0.046 ± 0.021
					Ye, 2013	T: 1.57 ± 0.66	T: 0.92 ± 0.42
						C: 1.58 ± 0.64	C: 1.24 ± 0.48
					Qiao, 2015	T: 1.52 ± 0.71	T: 0.91 ± 0.32
						C: 1.53 ± 0.81	C:1.22 ± 0.39
					Ma, 2018	T: 0.101 ± 0.027	T: 0.031 ± 0.027
						C: 0.098 ± 0.026	C:0.043 ± 0.022

*T, treatment group; C, control group*.

### Risk of Bias in Included Studies

**Figure 2:**
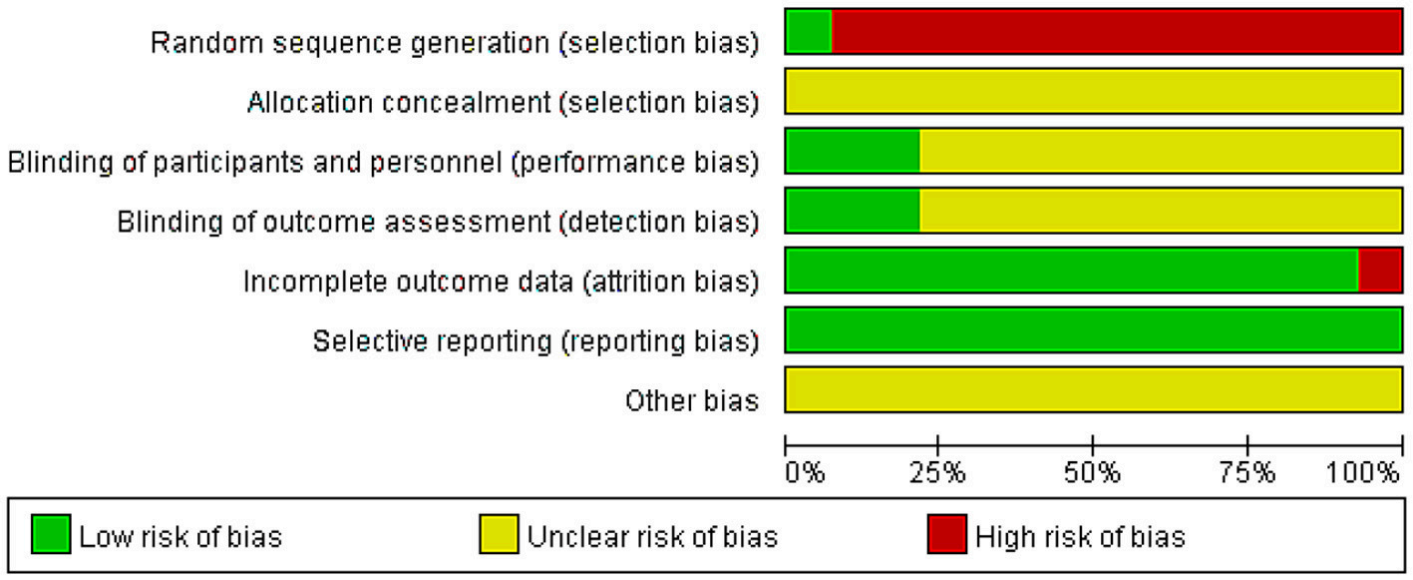
Risk of bias.

**Figure 3:**
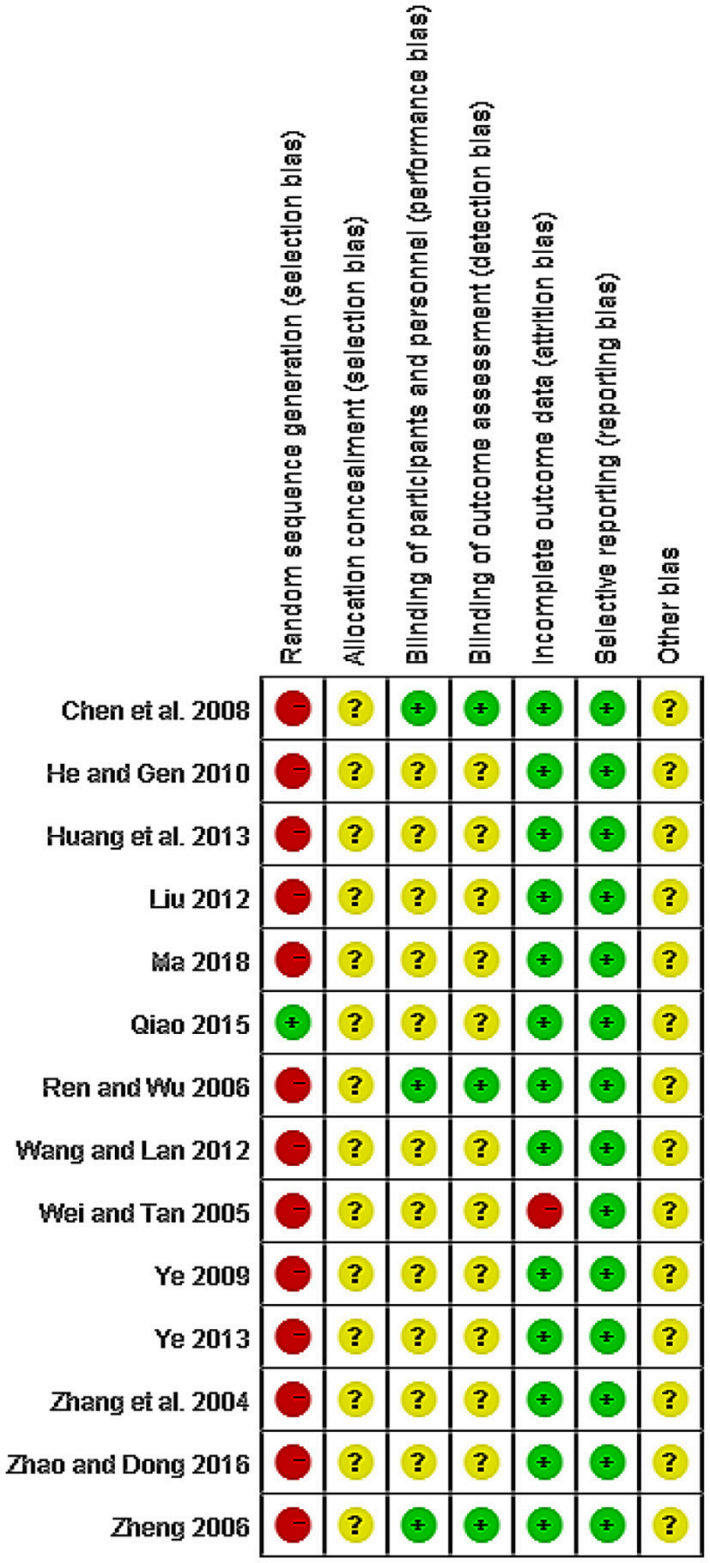
Risk of bias summary and graph.

The quality of all studies included was low. All studies were carried out among the Chinese population. Fourteen studies mention the use of random allocation: All studies failed to mention the specific grouping method, and none of the studies discussed allocation concealment, blinding, or evaluator blinding. The quality assessment is shown in Figures 2, 3 (Wu et al., 2018).

### Outcome Measures

#### Systolic Blood Pressure (SBP, mmHg)

**Figure 4:**
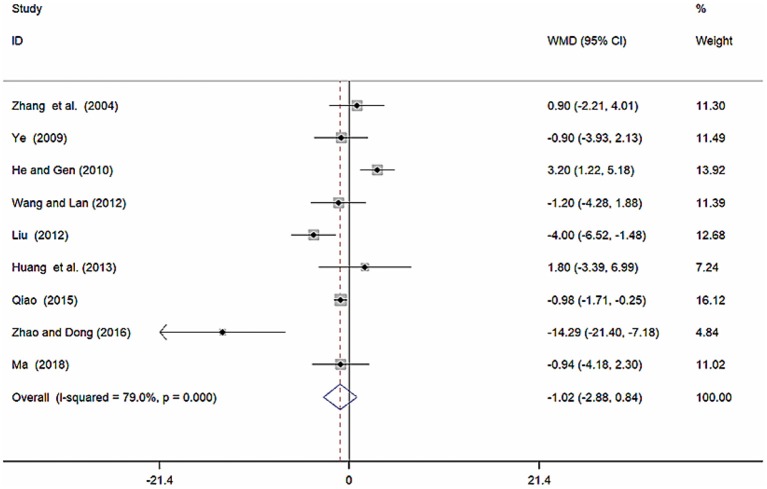
Forest plot of SBP of breviscapine injection plus antihypertensive drugs compared to antihypertensive drugs alone for hypertension-induced renal damage.

**Figure 5:**
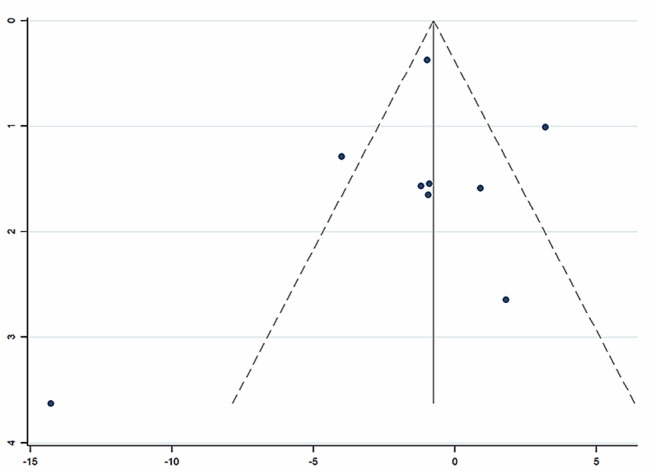
Funnel plot for the publication bias of SBP.

Nine studies (Zhang et al., 2004; Ye, 2009; He and Gen, 2010; Liu, 2012; Wang and Lan, 2012; Huang et al., 2013; Qiao, 2015; Zhao and Dong, 2016; Ma, 2018) involving 857 participants reported on the use of breviscapine injection plus antihypertensive drugs in the treatment of SBP for hypertension-induced renal damage. After the test for heterogeneity (*I*^2^ = 79.0%, *P* ≤ 0.001, Figure 4), we employed a random-effects model. A funnel plot analysis of the 9 trials suggested possible publication bias and inclusion of low quality studies as significant asymmetry is shown in Figure 5. We applied Egger's test to evaluate publication bias. A p (*P* = 0.757) value more than 0.05 was considered no publication bias (Supplementary Figure 1). The meta-analysis revealed that there was no significant difference between the experimental group and the control group in reducing SBP [WMD = −1.02, 95% CI (−2.88, 0.84), *P* = 0.281] (Supplementary Figure 2) (Wu et al., 2018).

#### Sensitivity Analysis of SBP

We conducted a sensitivity analysis for SBP (Supplementary Figure 3). By seriatim excluding one trial each time and re-performing meta-analysis of the remaining trials, we could observe whether the outcomes have dramatically changed. Sensitivity analysis indicated that the outcomes of Scr were very similar, which had relatively good stability.

#### Subgroup Analysis of SBP

**Figure 6:**
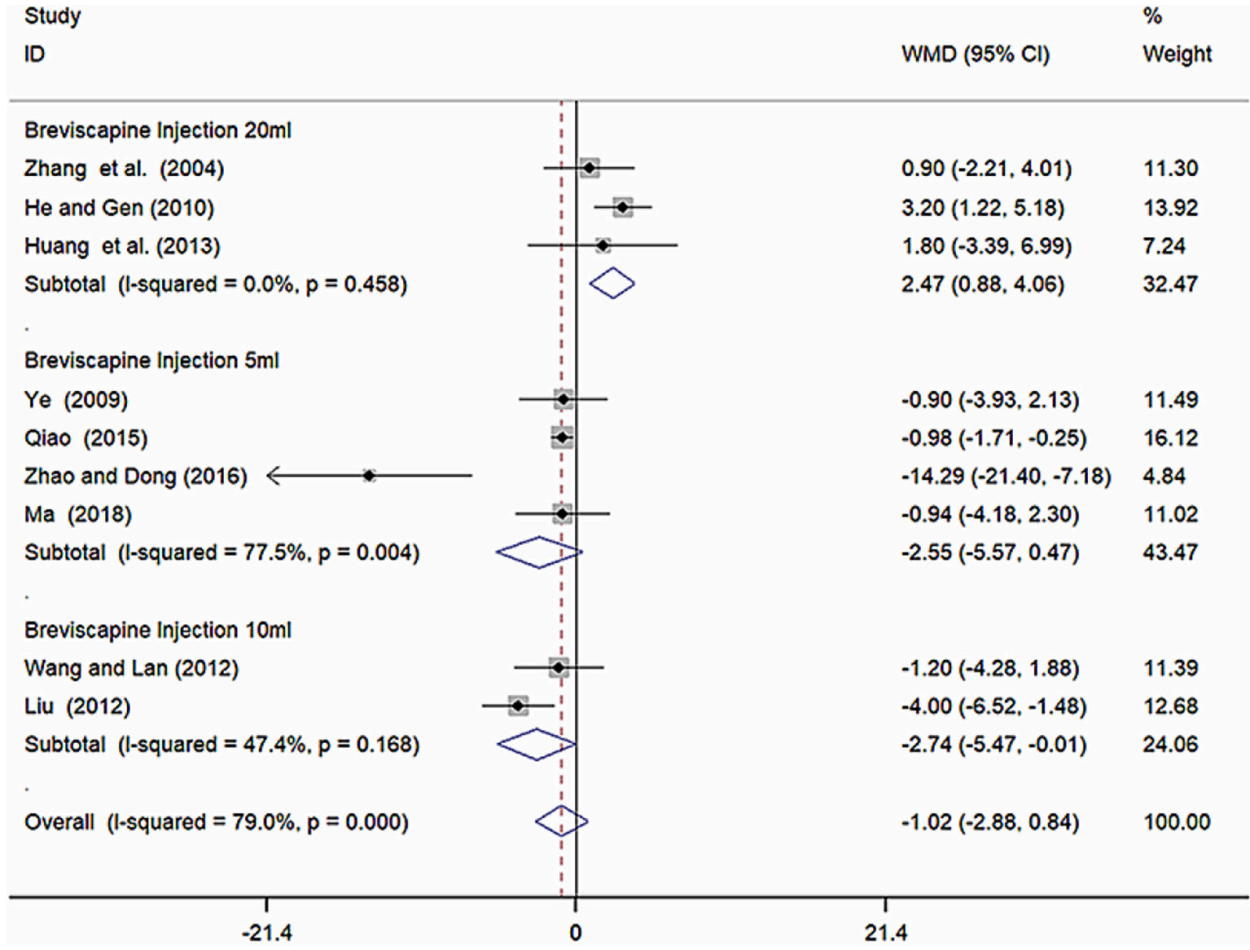
Subgroup analysis of different doses of breviscapine injection plus antihypertensive drugs compared to antihypertensive drugs alone in terms of SBP for hypertension-induced renal damage.

Because of high heterogeneity of SBP, we conducted subgroup analysis among studies using different different doses of breviscapine injection (20, 10, and 5 ml). Compared with the control groups, the results of subgroup analysis showed significant difference between breviscapine injection (20 ml) and antihypertensive drugs alone[WMD = 2.47, 95% CI (0.88, 4.06), *P* = 0.002], between breviscapine injection (10 ml) and antihypertensive drugs alone [WMD = −2.74, 95% CI (−5.47, −0.01), *P* = 0.002], but had no significant difference between breviscapine injection (5 ml) and antihypertensive drugs alone [WMD = −2.55, 95% CI (−5.57, 0.47), *P* = 0.097] (Figure 6, Supplementary Figure 4).

#### Diastolic Blood Pressure (DBP, mmHg)

**Figure 7:**
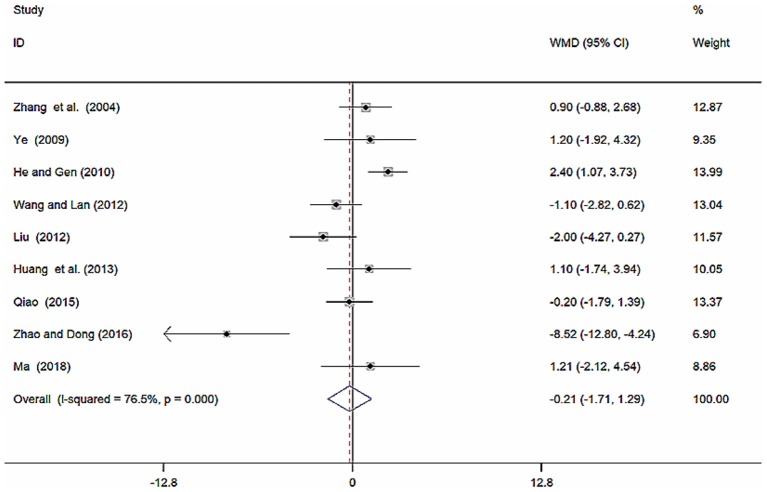
Forest plot of DBP of breviscapine injection plus antihypertensive drugs compared to antihypertensive drugs alone for hypertension-induced renal damage.

Nine studies (Zhang et al., 2004; Ye, 2009; He and Gen, 2010; Liu, 2012; Wang and Lan, 2012; Huang et al., 2013; Qiao, 2015; Zhao and Dong, 2016; Ma, 2018) involving 857 participants reported on the use of breviscapine injection plus antihypertensive drugs in the treatment of DBP for hypertension-induced renal damage. After the test for heterogeneity (*I*^2^ = 76.5%, *P* ≤ 0.001, Figure 7), we employed a random-effects model. We conducted a sensitivity analysis and applied Egger's test (*P* = 0.160) to evaluate publication bias for DBP (Supplementary Figures 5, 6). Meta-analysis showed a non-significant trend for reduction in DBP between the experimental group and the control group [WMD = −0.21, 95% CI (−1.71, 1.29), *P* = 0.786] (Supplementary Figure 7).

#### Subgroup Analysis of DBP

**Figure 8:**
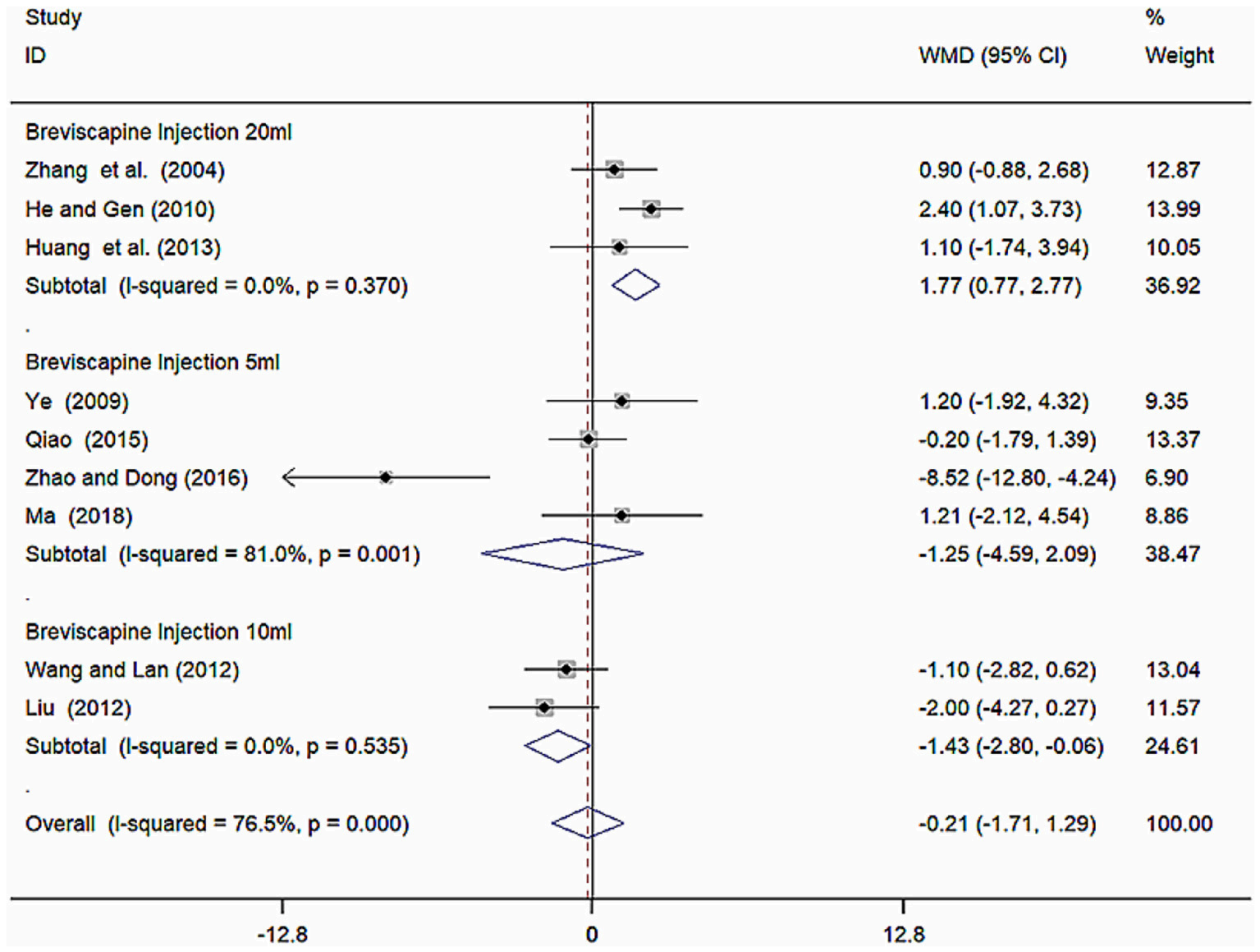
Subgroup analysis of different doses of breviscapine injection plus antihypertensive drugs compared to antihypertensive drugs alone in terms of DBP for hypertension-induced renal damage.

Because of high heterogeneity of DBP, we conducted subgroup analysis among studies using different different doses of breviscapine injection (20, 10, and 5 ml). Compared with the control groups, the results of subgroup analysis showed significant difference between breviscapine injection (20 ml) and antihypertensive drugs alone [WMD = 1.77, 95% CI (0.77, 2.77), *P* = 0.001], between breviscapine injection (10 ml) and antihypertensive drugs alone [WMD = −1.43, 95% CI (−2.80, −0.06), *P* = 0.041], but had non-significant difference between breviscapine injection (5 ml) and antihypertensive drugs alone [WMD = −1.25, 95% CI (−4.59, 2.09), *P* = 0.462] (Figure 8, Supplementary Figure 8).

#### Twenty-Four Hour Urinary Total Protein (24 h UTP, g/d)

**Figure 9:**
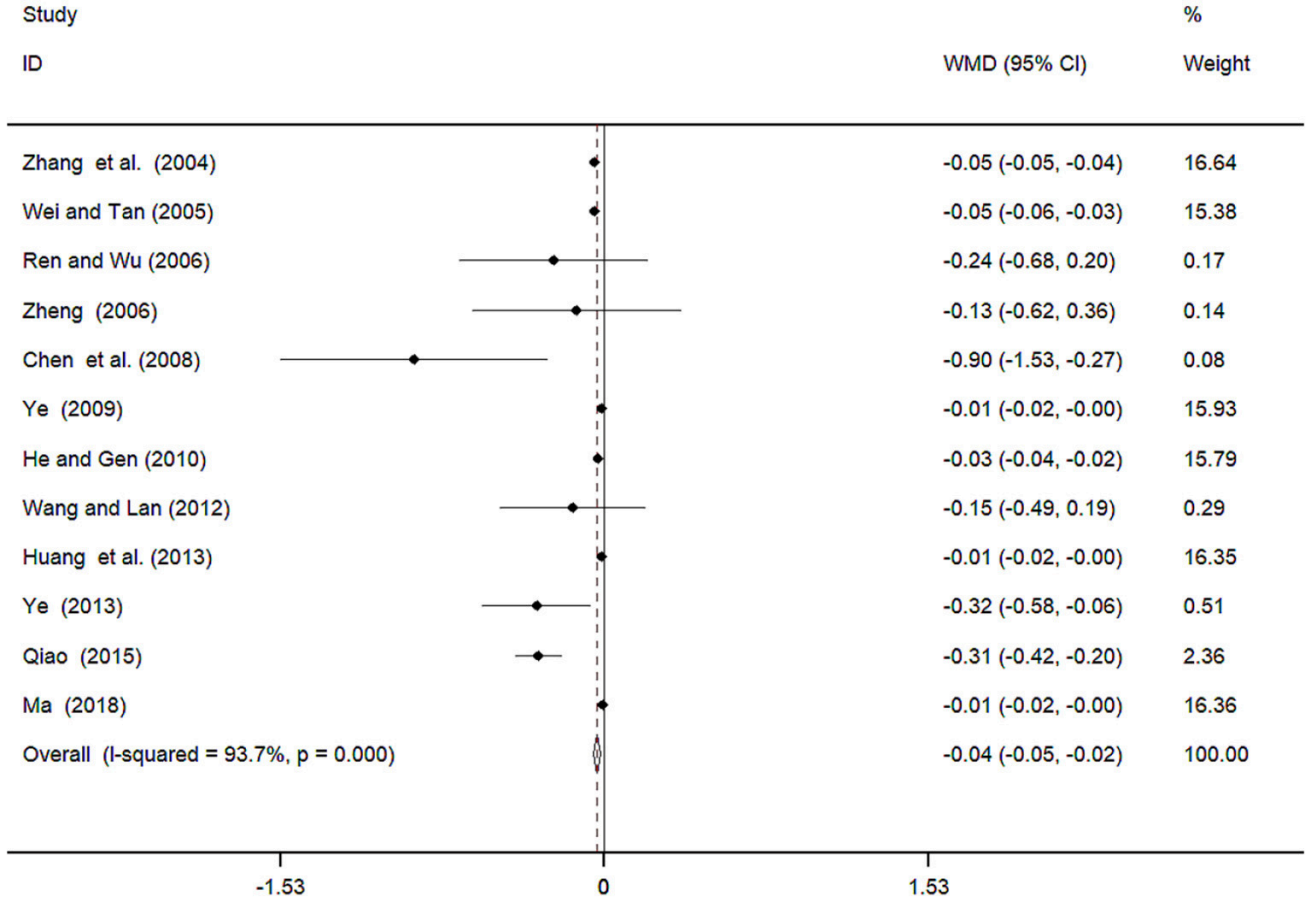
Meta-analyses results of breviscapine injection plus antihypertensive drugs compared to antihypertensive drugs alone in terms of the 24h UTP for hypertension-induced renal damage.

Twelve studies (Zhang et al., 2004; Wei and Tan, 2005; Ren and Wu, 2006; Zheng, 2006; Chen et al., 2008; Ye, 2009, 2013; He and Gen, 2010; Wang and Lan, 2012; Huang et al., 2013; Qiao, 2015; Ma, 2018) involving 941 participants reported on the use of breviscapine injection plus antihypertensive drugs in terms of the 24 h UTP for hypertension-induced renal damage. After the test for heterogeneity (*I*^2^ = 93.7%, *P* ≤ 0.001, Figure 9), we employed a random-effects model. We conducted a sensitivity analysis and applied the Egger's test (*P* = 0.586) to evaluate publication bias for 24 h UTP (Supplementary Figures 9, 10). The meta-analysis revealed that the experimental group performed better than the control group in reducing 24 h UTP [WMD = −0.04, 95% CI (−0.05, −0.02, *P* ≤ 0.001, Supplementary Figure 11) (Wu et al., 2018).

#### Adverse Effects

Nine of the included trials (Zhang et al., 2004; Wei and Tan, 2005; Ren and Wu, 2006; Zheng, 2006; Chen et al., 2008; Ye, 2009; He and Gen, 2010; Wang and Lan, 2012; Huang et al., 2013) described adverse effects in detail, while the others did not mention adverse events. Only one showed mild facial flushing during intravenous dripping of breviscapine (Wei and Tan, 2005), and after the speed of transfusion was slowed down, the symptom got remitted. Two cases (Huang et al., 2013) showed redness of limb skin in the experimental group. There were two cases (Ren and Wu, 2006; Zheng, 2006) of head inflation during the use of antihypertensive drugs. There were three cases (Chen et al., 2008; Ye, 2009) of head swelling, dizziness during infusionduring the use of breviscapine injection. Twenty-one cases showed dry cough due to the use of ACEI in six trials (Zhang et al., 2004; Ren and Wu, 2006; Zheng, 2006; Chen et al., 2008; He and Gen, 2010; Wang and Lan, 2012). None of the adverse events were serious. These symptoms are self-limited and did not affect the treatment.

## Discussion

This meta-analysis included 14 studies with 1,170 total participants comparing breviscapine injection plus antihypertensive drugs vs. antihypertensive drugs alone for hypertension in patients with hypertension-induced renal damage. Hypertension-induced renal damage marks with increased excretion of urinary protein, which is mostly attributed to increased permeability of glomerular filtration membrane, damage of electrostatic barrier, and renal tubular reabsorption. Urinary excretions of albumin and beta-2-microglobulin are sensitive to early functional damage of glomerular filtration and renal tubular reabsorption, respectively. It is defined as a urinary albumin excretion rate ranging from 30 to 300 mg/day, and the definitive measurement is based on a timed urine collection during a 24-h period. Evidence from previous systematic review shows that breviscapine injection in combination with antihypertensive drugs can improve creatinine clearance rate and reduce serum creatinine, blood urea nitrogen, 24-h urinary protein and beta-2-microglobulin in hypertensive nephropathy patients, a reduction in urinary protein may contribute toward the renal protective effect of breviscapine injection in hypertension-induced renal damage patients (Wu et al., 2018). This meta-analysis mainly explores the effects of breviscapine injection on hypertension in hypertension-induced renal damage patients.

Hypertension can affect each renal compartment: vessels, glomeruli and tubulointerstitium. Since the pioneering descriptions of nephrosclerosis by Theodor Fahr and Franz Volhard in 1918, a large number of studies have been published on hypertensive nephropathy (Meyrier, 2015; Seccia et al., 2017). Hypertension-induced renal damage is a common cause of ESRD and is associated with significant morbidity and mortality. To maximize reduction in the cardiovascular and renal mortality associated with hypertension-induced renal damage (Bakris, 2004; Kistorp et al., 2005), physicians and their patients should set specific treatment goals that must be achieved with the least intrusive strategy possible. The most effective strategy to maximally delay CKD progression must include albuminuria reduction and achievement of the BP goal of <130/80 mmHg (Whelton et al., 2018; Williams et al., 2018). This can be done by using multiple antihypertensive agents, including RAAS blockers and diuretics, together with adherence to dietary sodium restriction (Whelton et al., 2018; Williams et al., 2018).

Erigeron breviscapus is a kind of traditional Chinese medicine. It was first recorded in the book of “Yunnan Materia Medica.” According to Chinese medicine theory, erigeron breviscapus is cold-natured, sweet, bitter and pungent in taste, with the function of clearing heat, relieving toxicity, eliminating wind and dampness, activating blood and removing stasis, expediting channel and activating meridian, relieving inflammation, and alleviating pain. The monomer component of Chinese herbal medicine (CHM), also known as the natural pure compound drug, has recently attracted much attention. The natural extract artemisinin and its derivatives are good examples of monomer components of CHM that can treat diseases through various activities, and can be a good starting point to uncover the mechanism of traditional Chinese medicine.

Hypertension-induced renal damage is major target-organ damage due to sustained high BP. Long-term hypertension can cause renal sclerosis and gradually progress to chronic renal failure. Positive control of hypertension is the key to preventing hypertensive renal damage. It is unclear whether breviscapine can reduce blood pressure in animal studies or clinical trials, but breviscapine can improve myocardium in animal studies. Breviscapine could reverse the myocardial, interstitial and vascular remodeling, improve the rigidness of cardiac muscle, thus, has protective effect on heart. The mechanisms of their above-mentioned effects may be related to increase of cardiac myocyte apoptosis triggered by them via upregulation of apoptosis induced gene (Bax gene) expression and downregulation of apoptosis suppressor gene (Bcl-2 gene) expression as well as suppression of PKC activity (Li and Chen, 2002; Zhou et al., 2002). According to Chinese medicine theory, hypertensive renal injury is strongly related to fluid, phlegm and dampness retention syndrome and liver-yang hyperactivity syndrome, which are caused by deficiency syndrome. Chinese medicines, which are used to treat fluid, phlegm, and dampness retention syndrome, deficiency syndrome and liver-yang hyperactivity syndrome, respectively, have certain advantages with regard to treating hypertensive renal injury. Clinical research indicates that CHM might control increased SBP, inhibit the glomerular and tubular hyperplasia caused by high BP and significantly reduce urinary albumin and beta-2-microglobulin by increasing the activity of renal rennin and the level of Ang II (Shih et al., 2005; Yang et al., 2014, 2015; Xiong et al., 2015).

## Limitations

Several limiting factors in this study should be considered. First, the quality of the included randomized controlled trials was low. All included trials showed high or undefined risk of bias due to design, reporting, and methodology. There were no definitive, randomized, double-blind, placebo-controlled trials included in this meta-analysis. No trial reported detailed randomization methods, or allocation concealment. Therefore, “so-called” trials may not truly represent true randomized controlled trials, which may undermine the strength of clinical evidence and lead to potential selection bias. No trial was double-blinded, and unfortunately, none of the randomized-controlled trial has a placebo. Second, five trials did not report adverse reactions. Therefore, conclusions about safety cannot be made with confidence. Furthermore, certain active ingredients are chemically unstable, which limits large-scale synthesis. These pressing issues should be resolved in future research. The safety of breviscapine injection needs to be strictly monitored and properly reported in future clinical trials. Third, although most tests were conducted with small samples. We tried our best to avoid language bias and positional prejudice, but cannot rule out potential publication bias.

Overall, our results suggest that moderate dose of breviscapine injection (10 ml) has effects on reducing blood pressure in patients with hypertension-induced renal damage and high doses of breviscapine injection (20 ml) may increase blood pressure. More large-scale, multicenter, rigorously designed randomized controlled trials are needed to provide accurate data to further validate and s explore the mechanism of breviscapine injection for the treatment of hypertensive renal damage.

## Conclusions

Evidence from this systematic review shows that breviscapine injection in combination with antihypertensive drugs, can reduce 24-h urinary protein in patients with hypertension-induced renal damage, but overall have no effect on improving the blood pressure in hypertension-induced renal damage patients. Moderate dose of breviscapine injection (10 ml) may have effects on reducing blood pressure in hypertension-induced renal damage patients but high doses of breviscapine injection (20 ml) may increase blood pressure by subgroup analysis. However, the evidence of methodological quality and sample sizes is weak, and thus, further standardized research is required.

## Author Contributions

LW and ZF put forward this topic and designed this review. LW, YG, and SZ performed article screening, data collection and extraction, and manuscript writing. LW and YG conducted the data analysis.

### Conflict of Interest Statement

The authors declare that the research was conducted in the absence of any commercial or financial relationships that could be construed as a potential conflict of interest.
