# Supplementary material for: The Effects of Breviscapine Injection on Hypertension in Hypertension-Induced Renal Damage Patients: A Systematic Review and a Meta-Analysis
Source: Front Pharmacol. 2019 Feb 21;10:118. doi: 10.3389/fphar.2019.00118 (PMC6394135; doi:10.3389/fphar.2019.00118)
Supplement: Supplementary file 1 [file Table_1.DOCX]

**Supplementary Materials**

**Supplementary Tables S1 and S2 |** search strategy.

**Supplementary** **Figure S1 |** Egger’s test to evaluate publication bias of SBP.

**Supplementary** **Figure S2 |** Meta-analyses results of breviscapine injection plus antihypertensive drugs compared to antihypertensive drugs alone in terms of the SBP for hypertension-induced renal damage.

Supplementary **Figure S3 |** Sensitivity analysis for SBP.

Supplementary **Figure S4 |** Subgroup analysis of different doses of breviscapine injection plus antihypertensive drugs compared to antihypertensive drugs alone in terms of SBP for hypertension-induced renal damage.

Supplementary **Figure S5 |** Sensitivity analysis for DBP.

Supplementary **Figure S6 |** Egger’s test to evaluate publication bias of DBP.

Supplementary **Figure S7 |** Meta-analyses results of breviscapine injection plus antihypertensive drugs compared to antihypertensive drugs alone in terms of the DBP for hypertension-induced renal damage.

Supplementary **Figure S8 |** Subgroup analysis of different doses of breviscapine injection plus antihypertensive drugs compared to antihypertensive drugs alone in terms of DBP for hypertension-induced renal damage.

Supplementary **Figure S9 |** Sensitivity analysis for 24-hour urinary total protein.

Supplementary **Figure S10 |** Egger’s test to evaluate publication bias of 24-hour urinary total protein.

Supplementary **Figure S11 |** Meta-analyses results of breviscapine injection plus antihypertensive drugs compared to antihypertensive drugs alone in terms of the 24 h UTP for hypertension-induced renal damage.

**TABLE S1 |** Search strategies used for PubMed and other English language databases.

| #1 | Hypertension, Renal [MeSH Terms] OR “Hypertension, Renovascular” [Title/Abstract] OR “Hypertensive Nephropathy” [Title/Abstract] OR “Hypertensive Kidney Lesion” [Title/Abstract] OR “Hypertensive Renal Damage” [Title/Abstract] OR “Hypertension-induced renal damage” [Title/Abstract] |
| --- | --- |
| #2 | “Breviscapine” [Title/Abstract] OR “Breviscapine Injection” [Title/Abstract] OR “Dengzhanhua” [Title/Abstract] OR “Dengzhanhua Injection” [Title/Abstract] OR “BVP” [Title/Abstract] OR “BVP Injection” [Title/Abstract] |
| #3 | “Randomized controlled trial” [Title/Abstract] OR “Controlled clinical trial” [Title/Abstract] OR “Randomized” [Title/Abstract] |
| #4 | #1 AND #2 AND #3 |

**TABLE S2 |** Search strategies used for CNKI and other Chinese language databases.

| #1 | “Gao Xue Ya Shen Yan” [MeSH Terms] OR “Gao Xue Ya Shen Bing” [Title/ Abstract] OR “Gao Xue Ya Shen Shun Hai” |
| --- | --- |
| #2 | “Deng Zhan Hua” [MeSH Terms] OR “Dengzhanhua Preparations” [Title/Abstract] OR “Dengzhanhua Zhusheye” [Title/Abstract] OR “Dengzhanhua Zhusheye” [Title/Abstract] OR “Zhusheyong Dengzhanhua” [Title/Abstract] |
| #3 | “Suiji Duizhao Shiyan” [Title/Abstract] OR “Duizhao Linchuang Shiyan” [Title/Abstract] OR “Suiji” [Title/Abstract] |
| #4 | #1 AND #2 AND #3 |


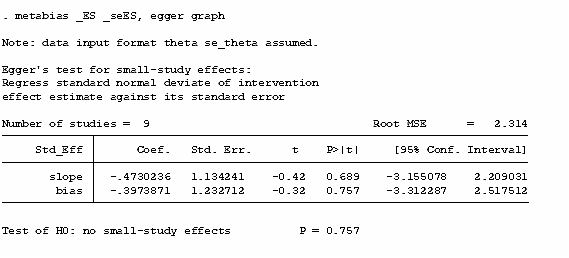


**FIGURE S1 |** Egger’s test to evaluate publication bias of SBP.


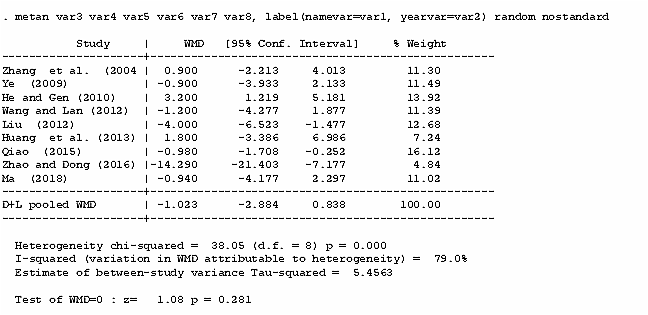


**FIGURE 2** **|** Meta-analyses results of breviscapine injection plus antihypertensive drugs compared to antihypertensive drugs alone in terms of the SBP for hypertension-induced renal damage.

.


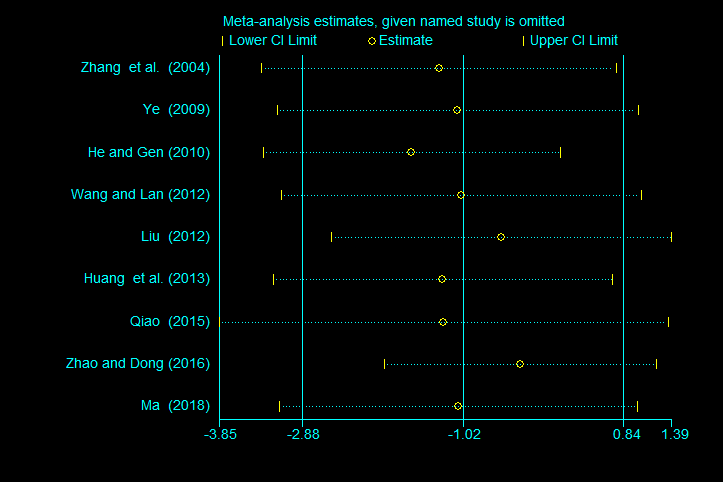


**FIGURE S3 |** Sensitivity analysis for SBP.


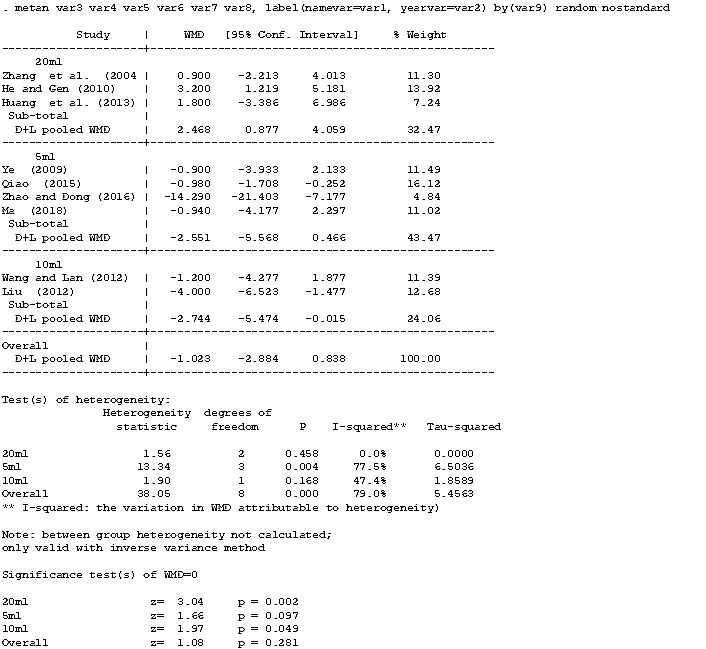


**FIGURE S4 |** Subgroup analysis of different doses of breviscapine injection plus antihypertensive drugs compared to antihypertensive drugs alone in terms of SBP for hypertension-induced renal damage.


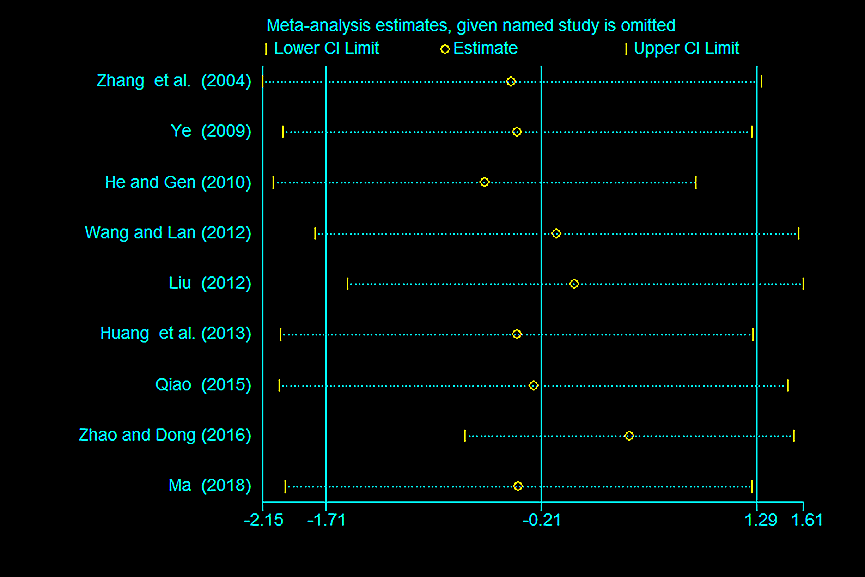


**FIGURE S5 | Sensitivity analysis for DBP.**


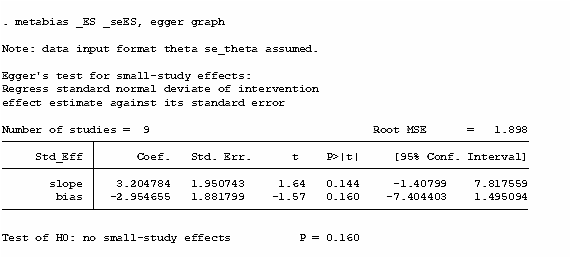


**FIGURE S6 |** Egger’s test to evaluate publication bias of DBP.


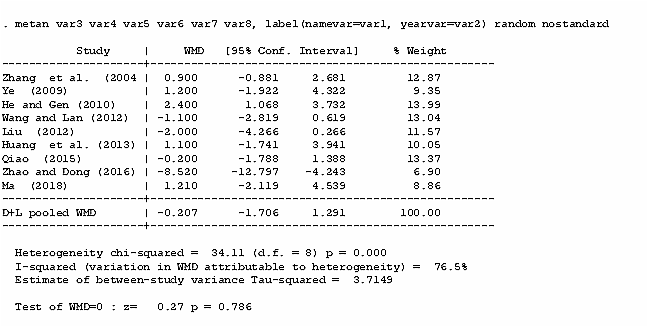


**FIGURE 7 |** Meta-analyses results of breviscapine injection plus antihypertensive drugs compared to antihypertensive drugs alone in terms of the DBP for hypertension-induced renal damage.


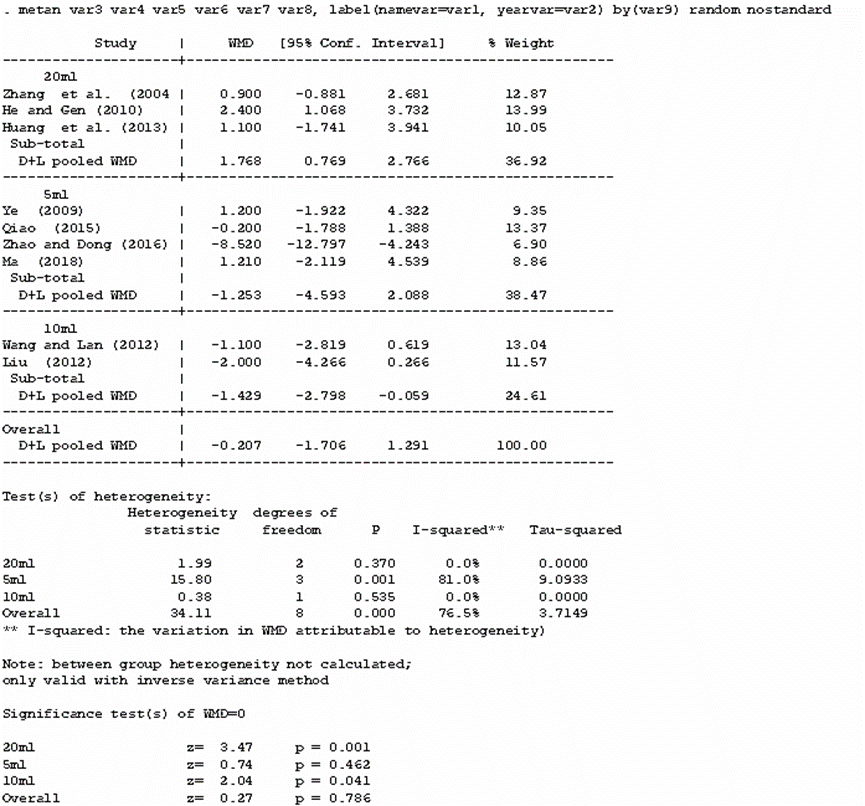


**FIGURE S8 |** Subgroup analysis of different doses of breviscapine injection plus antihypertensive drugs compared to antihypertensive drugs alone in terms of DBP for hypertension-induced renal damage.


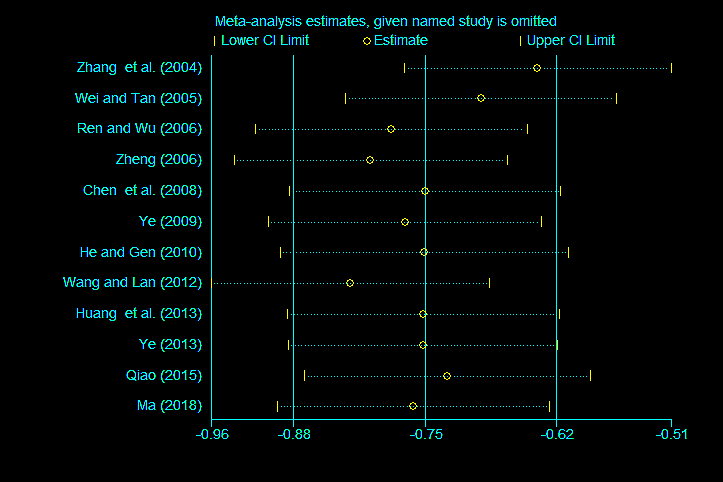


**FIGURE S9 |** Sensitivity analysis for 24-hour urinary total protein


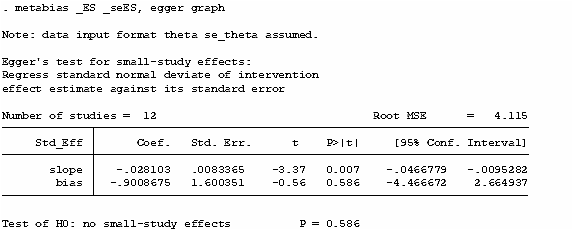


**FIGURE S10 |** Egger’s test to evaluate publication bias of 24-hour urinary total protein.


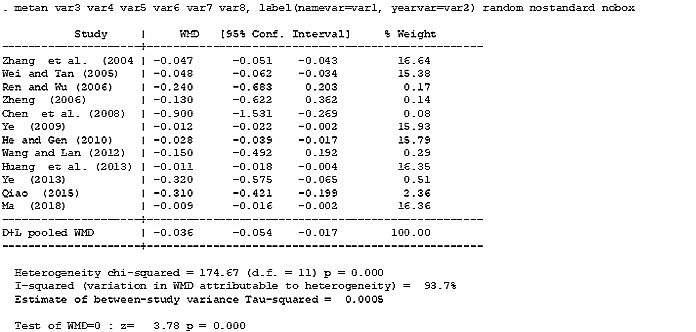


**FIGURE S11 |** Meta-analyses results of breviscapine injection plus antihypertensive drugs compared to antihypertensive drugs alone in terms of the 24 h UTP for hypertension-induced renal damage.
